# Mutations in the Genes for Interphotoreceptor Matrix Proteoglycans, *IMPG1* and *IMPG2*, in Patients with Vitelliform Macular Lesions

**DOI:** 10.3390/genes8070170

**Published:** 2017-06-23

**Authors:** Caroline Brandl, Heidi L. Schulz, Peter Charbel Issa, Johannes Birtel, Richard Bergholz, Clemens Lange, Claudia Dahlke, Ditta Zobor, Bernhard H. F. Weber, Heidi Stöhr

**Affiliations:** 1 Klinik und Poliklinik für Augenheilkunde, Universitätsklinikum Regensburg, 93053 Regensburg, Germany; Caroline.Brandl@ukr.de; 2 Institut für Humangenetik, Universität Regensburg, 93053 Regensburg, Germany; Heidi.Schulz@klinik.uni-regensburg.de (H.L.S.); Bernhard.Weber@klinik.uni-regensburg.de (B.H.F.W.); 3 Lehrstuhl für Genetische Epidemiologie, Universität Regensburg, 93053 Regensburg, Germany; 4 Department of Ophthalmology, University of Bonn, 53113 Bonn, Germany; Johannes.Birtel@ukbonn.de; 5 Oxford Eye Hospital, OUH NHS Foundation Trust and the Nuffield Laboratory of Ophthalmology, Department of Clinical Neurosciences, University of Oxford, Oxford OX1 3BD, UK; 6 Klinik für Augenheilkunde, Charité—Universitätsmedizin Berlin, 10117 Berlin, Germany; Richard.Bergholz@charite.de; 7 Klinik für Augenheilkunde, Universitätsklinikum Freiburg, Medizinische Fakultät, Albert Ludwigs Universität Freiburg, 79085 Freiburg, Germany; Clemens.Lange@uniklinik-freiburg.de; 8 Klinik für Augenheilkunde, Universitätsklinikum Köln, 50937 Köln, Germany; Claudia.Dahlke@uk-koeln.de; 9 Forschungsinstitut für Augenheilkunde, Universitätsklinikum Tübingen, 72076 Tübingen, Germany; ditta.zobor@uni-tuebingen.de

**Keywords:** vitelliform macular dystrophy, *IMPG1*, *IMPG2*, interphotoreceptor matrix, optical coherence tomography

## Abstract

A significant portion of patients diagnosed with vitelliform macular dystrophy (VMD) do not carry causative mutations in the classic VMD genes *BEST1* or *PRPH2*. We therefore performed a mutational screen in a cohort of 106 *BEST1/PRPH2*-negative VMD patients in two genes encoding secreted interphotoreceptor matrix proteoglycans-1 and -2 (*IMPG1* and *IMPG2*). We identified two novel mutations in *IMPG1* in two simplex VMD cases with disease onset in their early childhood, a heterozygous p.(Leu238Pro) missense mutation and a homozygous c.807 + 5G > A splice site mutation. The latter induced partial skipping of exon 7 of *IMPG1* in an in vitro splicing assay. Furthermore, we found heterozygous mutations including three stop [p.(Glu226*), p.(Ser522*), p.(Gln856*)] and five missense mutations [p.(Ala243Pro), p.(Gly1008Asp), p.(Phe1016Ser), p.(Tyr1042Cys), p.(Cys1077Phe)] in the *IMPG2* gene, one of them, p.(Cys1077Phe), previously associated with VMD. Asymptomatic carriers of the p.(Ala243Pro) and p.(Cys1077Phe) mutations show subtle foveal irregularities that could characterize a subclinical stage of disease. Taken together, our results provide further evidence for an involvement of dominant and recessive mutations in *IMPG1* and *IMPG2* in VMD pathology. There is a remarkable similarity in the clinical appearance of mutation carriers, presenting with bilateral, central, dome-shaped foveal accumulation of yellowish material with preserved integrity of the retinal pigment epithelium (RPE). Clinical symptoms tend to be more severe for *IMPG1* mutations.

## 1. Introduction

The interphotoreceptor matrix proteoglycans-1 and -2, also known as sialoprotein associated with cones and rods (SPACR) and SPACRCAN (a proteoglyCan related to SPACR), respectively, are large glycosylated protein components of the insoluble interphotoreceptor matrix (IPM) [1]. The IPM is a specialized type of extracellular matrix that surrounds the photoreceptor inner and outer segments and the apical processes of the retinal pigment epithelium (RPE). It serves several important functions, including communication between photoreceptors, RPE and Müller cells, by regulating trafficking of signaling molecules, nutrients and metabolites and the maintenance of retinal adhesion and photoreceptor alignment [1,2] IMPG1 and IMPG2 have hyaluronan (HA) binding motifs of the receptor for HA-mediated motility (RHAMM) type [1,3]. It is believed that secreted IMPG1 and IMPG2 bind HA and that this interaction stabilizes or modulates the IPM scaffold [1].

First evidence for an involvement of the interphotoreceptor matrix proteoglycans in the etiology of retinal degenerative disease was given when the heterozygous c.1736T > C/p.(Leu579Pro) mutation in the *IMPG1* gene was shown to cause autosomal dominant benign concentric annular macular dystrophy (BCAMD) in a Dutch family [4]. This phenotype is characterized by an initial parafoveal hypopigmentation and good visual acuity which then progresses to a retinitis pigmentosa-like manifestation. Subsequently, autosomal recessive mutations in the *IMPG2* gene were identified to be causally associated with early-onset retinitis pigmentosa (RP) [5]. The RP phenotype is frequently accompanied by early macular abnormalities, ranging from minor pigment alterations to profound chorioretinal atrophy [6].

Recently, mutations in *IMPG1* and *IMPG2* have been reported to also play a causative role in autosomal dominant and recessive vitelliform macular dystrophy (VMD) [7,8]. Vitelliform lesions have been defined by Gass in 1974 as bilateral, round or oval, yellow, symmetrical, singular, subretinal lesions, typically one-third to one-half disc diameter in size [9]. Best disease is caused by dominant (mostly missense) mutations in the *BEST1* gene [10,11]. Characteristic features of this disease entity include a diminished Arden ratio measured by electrooculography (EOG) as well as the typical egg yolk-like vitelliform macular lesion, which can already occur during childhood [12]. In contrast, adult-onset VMD (AVMD) becomes symptomatic not before the fourth or fifth decade of life with a relatively mild and slowly progressive loss of central visual acuity [12,13]. Another main clinical aspect in AVMD, and an important difference to Best disease, is a normal or only slightly subnormal light induced rise in ocular potential [12,14]. AVMD is genetically more heterogeneous than Best disease and has in some instances been linked to dominant mutations in *BEST1* but also in the *PRPH2* gene [15].

In the present study, we aimed to further investigate the role of *IMPG1* and *IMPG2* sequence variations in the development of vitelliform macular disease in our large patient cohort. We have screened the *IMPG1* and *IMPG2* genes in a total of 106 unrelated patients diagnosed with Best disease or AVMD but found negative after *BEST1* or *PRPH2* mutation testing. We identified two patients with novel causative mutations in the *IMPG1* gene. In addition, three novel stop mutations and four novel missense mutations as well as the c.3230G > T/p.(Cys1077Phe) mutation reported before [8] were found in the *IMPG2* gene to be heterozygously present in eight patients. To assess the pathogenicity of these mutations we performed bioinformatic evaluation, in vitro splice assays, database searches and family segregation as well as comparative clinical characterization of the patients. 

## 2. Subjects and Methods 

### 2.1. Patient Recruitment

The study included a total of 106 patients with suspected VMD or AVMD who underwent routine genetic testing at the Institute of Human Genetics Regensburg and which were tested negative for mutations in the coding exons/flanking intronic sequences of *BEST1* and *PRPH2*. A positive family history of VMD was reported for 15 cases, 46 individuals had a negative family history and no further information on the familial status of retinal degeneration was available for 45 patients. Seven unaffected family members were available for carrier testing. In accordance with the German Genetic Diagnostics Act written informed consent for genetic analysis was obtained from all subjects. This study adhered to the tenets of the Declaration of Helsinki. The study was also approved by the Ethics Committee of the University Regensburg, Germany (ID 17-514-104, date of approval 24.03.2017).

### 2.2. Clinical and Functional Ophthalmological Evaluation

Clinical, imaging, and electrophysiological findings were available and reviewed for ten index patients carrying mutations in the *IMPG1* or *IMPG2* gene as well as for seven family members. Age at initial diagnosis corresponds to the patient´s first visit to the respective University eye clinic in Regensburg, Bonn, Freiburg, Köln, Tübingen and Berlin, where the diagnosis of vitelliform macular dystrophy was established. Best-corrected visual acuity (VA) was assessed with standard charts under standard clinic conditions and results were transferred into logarithm of the Minimum Angle of Resolution (logMAR). 

Electrooculograms (EOG) were recorded in six of ten index patients according to the standards of the International Society of Clinical Electrophysiology of Vision (ISCEV) [16]. A light peak-to-dark trough ratio of <1.5 was defined as abnormally low, of >2.0 as normal, of between 1.5 and 2.0 as borderline, in line with the ISCEV recommendations [16]. Moreover, in six of ten patients, multifocal electroretinograms (mfERG) were conducted following ISCEV standards [17].

Multimodal retinal imaging included color fundus photography, obtained with the Zeiss FF450+ or Visucam (Carl Zeiss Meditec AG, Jena, Germany) and spectral domain-optical coherence tomography (SD-OCT) as well as fundus autofluorescence (FAF) imaging, performed with a combined Heidelberg Retina Angiograph and SD-OCT Spectralis device (Heidelberg Engineering, Heidelberg, Germany).

### 2.3. Molecular Analysis

For DNA extractions, ethlylenediaminetetraacetic acid (EDTA) peripheral blood samples were obtained from all patients and family members. Next-generation sequencing (NGS) was performed using the ION Torrent^TM^ semiconductor technology (Thermo Fisher Scientific, Dreieich, Germany) upon multiplex-PCR amplification of the gene fragments by exon flanking oligonucleotide primer pairs (sequences available upon request) using the QIAGEN Multiplex PCR Master Mix (Qiagen, Hilden, Germany). In brief, the multiplexed PCR reactions of each patient were pooled after quantification in the MultiNA Shimadzu system (Shimadzu Biotech, Duisburg, Germany) and processed using the Ion Xpress™ Plus Fragment Library kit (Thermo Fisher Scientific, Dreieich, Germany) according to the manufacturers recommendations. The barcoded DNA libraries were purified with AMPure beads (Beckman Coulter, Krefeld, Germany) and the resulting library concentration was determined using an Agilent 2100 bioanalyzer (Agilent, Waldbronn, Germany). The emulsion PCR was carried out on the Ion OneTouch™ 2 System and the samples were loaded on a 318 Ion Torrent™ System Chip and sequenced with an ION Personal Genome Machine (Thermo Fisher Scientific, Dreieich, Germany). NGS data were analyzed with the CLC Genomics Workbench (CLC bio, Aarhus, Denmark). The mean coverage for the amplicons ranged from 56× to 5420×, with all bases sequenced at least 34×. Potentially disease-relevant variants were confirmed by Sanger chain-terminating dideoxynucleotide sequencing.

### 2.4. Bioinformatics Analysis

Variants were classified as recommended by the American College of Medical Genetics and Genomics (ACMG) standards and guidelines and the Association for Molecular Pathology (AMP) Clinical Practice Guidelines. The recommendations were based on population data, computational data and previous publications. Variant frequencies were taken from the Browser of the Exome Aggregation Consortium (ExAC), variants with a minor allele frequency (MAF) > 0.005 were excluded from further analysis. Rare missense mutations were tested for disease relevance by prediction algorithms of the programs MutationTaster, SIFT and PolyPhen-2. When at least two of the three programs predicted a pathogenic effect, the variants were classified as “likely pathogenic”. Stop mutations were classified as pathogenic. Intronic and synonymous variants were further analyzed for a potential effect on correct splicing by using the interface provided by the software package Alamut (Interactive Biosoftware, Rouen, France, Alamut Visual 2.7.1) which combines five algorithms (SpliceSiteFnder, MaxEntScan, NNSPLICE, GeneSplicer and Human Splicing Finder). All tools provide prediction scores for the normal and the mutant allele.

### 2.5. Minigene Assay

Functional consequences of *IMPG1* splicing variant c.807 + 5G > A was analyzed with the pSPL3b-based exon trapping system. Briefly, exon 7 of the *IMPG1* gene was PCR amplified from genomic DNA of patient #2 and control using a 7:1 ratio of Taq:Pfu polymerase and oligonucleotide primer pairs flanked with restriction sites for *Not*I and *BamH*I (5’-GCG GCC GCG CCT TCA TAA TCC ACT TCT TGA-3’; 5’-GGA TCC GGG CCC ATT GTA ATT TTG GT-3’) and cloned into the pSPL3b vector (kindly provided by Dr. M. Gessler, University Würzburg, Germany). HEK293 cells were transfected in duplicate with 3 µg of construct DNA using TransIT^®^-LT1 Transfection Reagent (Mirus, Madison, WI, USA), harvested after 48 hours and total RNA was extracted using RNeasy Micro Kit (Qiagen, Hilden, Germany). RT-PCR was performed with the RevertAid™ H First Strand cDNA Synthesis Kit (Thermo Fisher Scientific, Waltham, MA, USA) and a pSPL3-specific SD6 (5’-TCT GAG TCA CCT GGA CAA CC-3’) and SA4 (5’-CAC CTG AGG AGT GAA TTG GTC G-3’) primer pair in combination with oligonucleotide primers for the housekeeping gene ACTB (5’-GAC ATC CGC AAA GAC CTG TA-3’; 5’-CAG GAG AGC AAT GAT CTT GA-3’). PCR products were separated by electrophoresis on a 2% agarose gel, excised, cloned and Sanger sequenced.

## 3. Results

### 3.1. Patients with Mutations Identified in the IMPG1 Gene

Mutational analysis of the *IMPG1* gene identified putative causative mutations in two families (Table 1). Patient #1 of the German family 13-501 carries a heterozygous c.713T > C nucleotide change in exon 7 of the *IMPG1* gene that leads to a p.(Leu238Pro) amino acid substitution in the SEA I domain (Sperm protein, Enterokinase and Agrin) of the IMPG1 protein and is predicted to be pathogenic by all three algorithms used in the in silico analysis. At the age of 13, patient #1 showed reduced visual acuity with 0.2 and 0.1 logMAR on the right and left eye, respectively. The fundus of both eyes revealed central RPE abnormalities with foveal detachment (images not available). Patient #1 is the only affected member in the family. Data on ophthalmological examination or genetic testing of the parents of patient #1 were not available.

**Figure 1:**
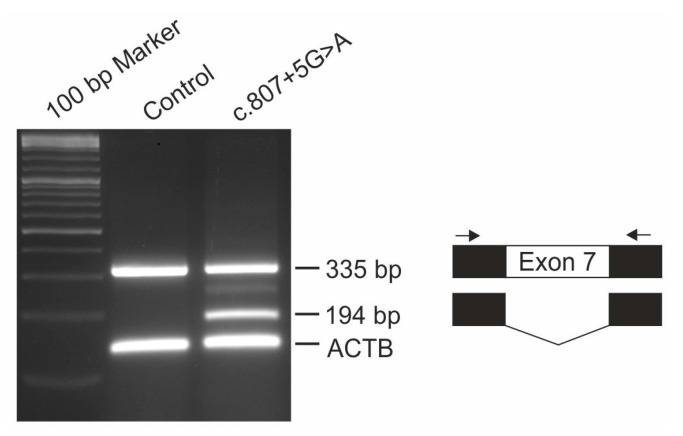
In vitro splicing assay of the *IMPG1* mutation c.807 + 5G > A. Genomic DNA fragments containing exon 7 and flanking intronic sequences from patient #2 carrying the c.807 + 5G > A mutation and a control individual were cloned into pSPL3b and overexpressed in HEK293 cells. RNA isolated from these cells was reverse transcriptase (RT)-PCR amplified using pSPL3b-specific primers SD6 and SA4 (indicated by arrows). Schematic illustration of the splice products separated by gel electrophoresis is depicted on the right. The c.807 + 5G > A mutation leads to partial skipping of *IMPG1* exon 7. RT-PCR amplification of ACTB served as control.

A second *IMPG1* mutation, c.807 + 5G > A, was found in patient #2 of the consanguineous Turkish family 11-151 in a homozygous state (Table 1). Homozygosity was confirmed by segregation analysis in the genomic DNA of the parents. The intronic c.807 + 5G > A nucleotide substitution resides within in the conserved 5’ splice site of exon 7 and is predicted to weaken the donor splice site by 20%. The analysis of cDNA derived from HEK293 cells transfected with a construct containing the c.807 + 5G > A mutation, revealed aberrant pre-mRNA splicing; exon 7 was skipped in about half of the transcripts leading to a 194 bp as well as the 335 bp reverse transcription (RT)-PCR product amplified in controls (Figure 1). An additional faint band of ~270 bp of unknown origin was seen in cells transfected with the mutant construct. The in vitro splice assay thus confirms the bioinformatic prediction of missplicing due to the c.807 + 5G > A variant that likely leads to nonsense-mediated mRNA decay (NMD) of the aberrant mRNA.

**Figure 2:**
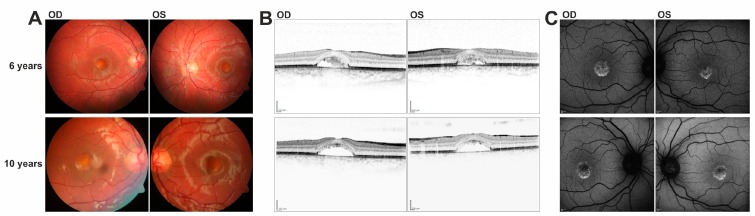
Multimodal retinal imaging in patient #2 with homozygous c.807 + 5G > A mutation in *IMPG1*. (**A**) Color fundus images of right (OD) and left (OS) eye acquired at the age of six and ten years reveal bilateral, yellowish vitelliform lesions with a granular aspect and no satellite drusen, which seem stable during these years of follow-up. (**B**) Corresponding horizontal SD-OCT scans through the fovea at the age of 6 years demonstrate a central, dome-shaped foveal detachment of the neurosensory retina with some solid material underneath. The latter is resorbed during follow-up, leading to an optically empty cavity at the age of ten. Photoreceptor outer segments seem to be elongated. Bruch’s membrane and RPE maintain classical reflectivity and seem well preserved. (**C**) Corresponding FAF images show bilateral, central, circular hyperfluorescence, which decreases during follow-up (in line with the resorption of the solid material).

Patient #2 was initially diagnosed with vitelliform macular dystrophy at the age of 2 (Table 2). During an eight year follow-up period the boy demonstrated fluctuating visual acuity of ~0.2 logMAR in both eyes. Electrophysiology at the age of 10 revealed normal findings in mfERG and a borderline to slightly abnormally low Arden Ratio in EOG measurements (Table 2). Color fundus images showed a singular, vitelliform lesion in the central macula of both eyes, but otherwise normal fundus features (Figure 2A). The area of the vitelliform lesion increased over the 4-year follow-up period and corresponding SD-OCT scans revealed a bilateral, central, dome-shaped foveal detachment of the neurosensory retina (Figure 2B). Hyperreflective material underneath was reabsorbed during follow-up and has left behind an optically empty cavity. Photoreceptors demonstrated an elongation of outer segments. Bruch’s membrane and RPE maintained its classical reflectivity and seemed well preserved (Figure 2B). Corresponding FAF imaging showed circular hyperfluorescence which decreased in the course of disease (Figure 2C). Both heterozygous 35- and 40-year-old parents of patient #2 are clinically asymptomatic with full visual acuity and regular findings in multimodal retinal imaging (data not shown).

### 3.2. Patients with Mutations Identified in the IMPG2 Gene

**Figure 3:**
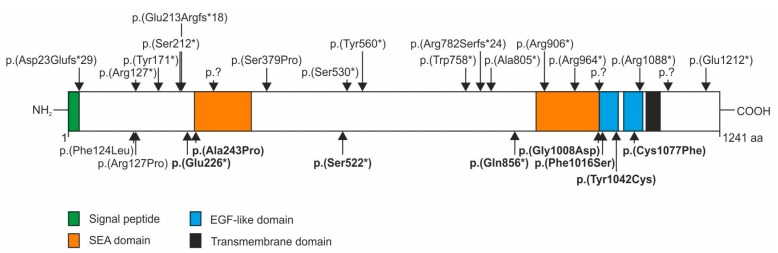
Schematic representation of the IMPG2 protein and mutations. The IMPG2 protein is depicted as a white bar with the respective protein domains indicated in different colours. Mutations identified in retinitis pigmentosa patients are shown above, mutations identified in patients with other forms of retinal dystrophy are shown below the IMPG2 protein. Those in bold are mutations identified in VMD patients described in this study. Mutations with an expected effect on the protein level but without a reliable prediction of the consequence (e.g., splice mutations) are indicated as p.?.

Eight patients were found to carry heterozygous mutations in the *IMPG2* gene including three stop mutations [p.(Glu226*), p.(Ser522*) and p.(Gln856*)] and five missense mutations [p.(Ala243Pro), p.(Gly1008Arg), p.(Phe1016Ser), p.(Tyr1042Cys) and p.(Cys1077Phe)] (Figure 3). The latter missense mutations all have strong in silico evidence to affect IMPG2 protein function (Table 1). Except for mutation c.3023G > A/p.(Gly1008Asp), which was found once in 120.878 control alleles, none of the other seven sequence changes is listed in the ExAC database (Table 1).

**Figure 4:**
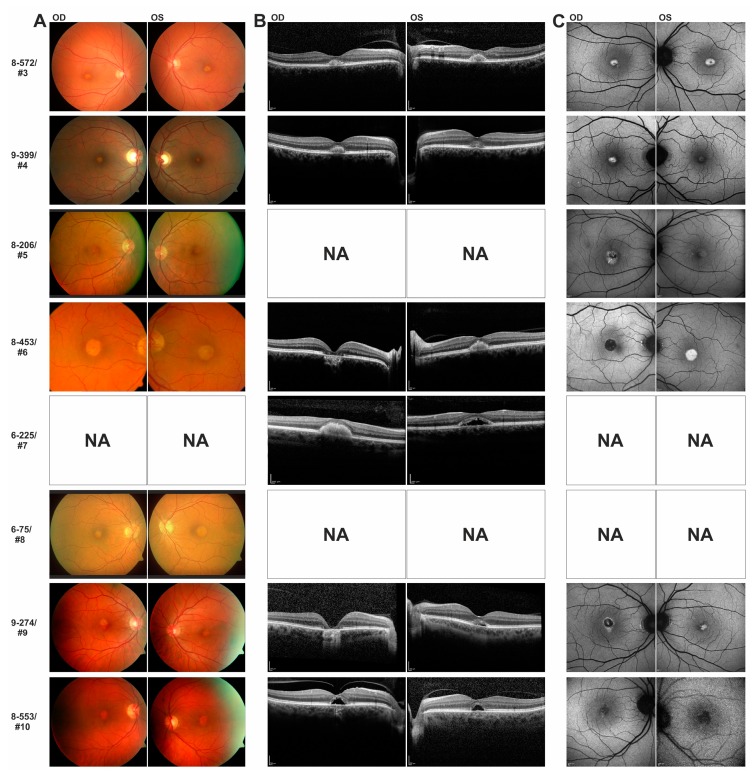
Multimodal retinal imaging of eight VMD patients with heterozygous mutations in *IMPG2*. Patients have been sorted by ascending cDNA position of the respective mutation (see Table 1). (**A**) Color fundus images of right (OD) and left (OS) eyes reveal bilateral, round, yellowish vitelliform lesions of different sizes with a granular aspect and no satellite drusen, or central atrophy (#6 OD and #9 OD). (**B**) Corresponding SD-OCT visualizes foveal detachment of the neurosensory retina with hyperreflective material (corresponding to the vitelliform material) located above the seemingly preserved Bruch’s membrane/RPE (#3 both eyes, #4 both eyes, #6 OS, #7 OD). The solid material is in some cases partially or fully resorbed, leaving behind a dome-shaped optically empty cavity (#7 OS, #9 OS, #10 both eyes) or central atrophy (#6 OD, #9 OD). (**C**) FAF shows either central hyperfluorescence corresponding to the vitelliform material (#3 both eyes, #4 both eyes, #5 both eyes, #6 OS, #9 OS), or hypofluorescence corresponding to an optically empty cavity (#10 both eyes) or central atrophy (#6 OD, #9 OD). NA = images not available.

For all eight index patients carrying heterozygous *IMPG2* mutations clinical information was available. All individuals were symptomatic, reported a slowly progressing central vision loss over years and did not receive any specific ocular treatment. Mean age at initial diagnosis was 53.1 years (range 33–64 years, Table 2), only one patient (#7) was younger than 45 years at initial diagnosis. Mean visual acuity was 0.4 logMAR in the right eye (range 0.7–0.1) and 0.2 logMAR in the left eye (range 0.3–0.1). All patients revealed visual acuity ≤0.3 logMAR in the better eye (Table 2). Electrophysiological findings were available for seven of the eight patients (Table 2). The light peak-to-dark trough ratio measured by EOG was borderline in one and within the normal range in three patients. Multifocal ERG measurements revealed normal findings in one patient (#7), the remaining four patients showed reduced amplitudes in the central rings of at least one eye (Table 2). Patient #4 had regular full-field ERG measurements available. Multimodal retinal imaging via color fundus photography, SD-OCT and FAF revealed highly similar findings in all eight index patients (Figure 4).

One set of eyes (both eyes of #3, #4, #5 and #8, #6 left eye, #7 right eye) showed differently sized, singular, brown-yellowish vitelliform lesions in the central macula on color fundus images (Figure 4A). Corresponding SD-OCT scans (when available), visualized a foveal detachment of the neurosensory retina with hyperreflective material located above the seemingly preserved Bruch’s membrane/RPE (Figure 4B). Matching FAF images demonstrated hyperfluorescence (Figure 4C). Both, SD-OCT and FAF findings corresponded to the vitelliform material seen on color fundus images. 

In a second set of eyes (#10 both eyes, #7 and #9 left eyes), the subretinal material was partially or completely resorbed, leaving behind a dome-shaped, optically empty cavity underneath the elevated neurosensory retina in SD-OCT scans (Figure 4B). Bruch’s membrane/RPE was still well preserved and maintained its classical reflectivity. A corresponding enlarged central hypofluorescence was observed by FAF (Figure 4C). 

Finally, in a third set of eyes, color fundus images as well as SD- OCT scans demonstrated central atrophy with loss of RPE in the right eyes of patients #6 and #9 which matched hypofluorescence seen by FAF (Figure 4A–C). As expected, visual acuity in these two eyes was markedly reduced to 0.6 logMAR (Table 2). Disease severity as defined by age of onset, visual acuity or lesions seen by retinal imaging was not associated with a specific type of mutation (nonsense versus missense mutation) in our patients. Importantly, associated drusen-like lesions were not detectable in the foveal zone or at the posterior pole.

### 3.3. Ophthalmological and Genetic Analysis of Relatives of Patients with IMPG2 Mutations

**Figure 5:**
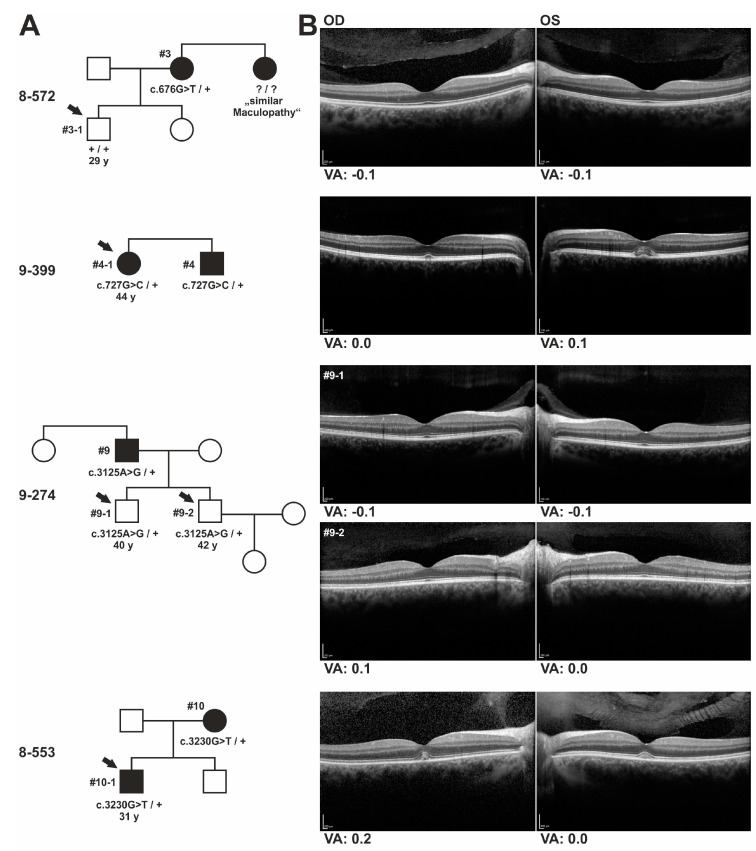
Genetics and clinical data of family members of VMD patients with heterozygous *IMPG2* mutations. (**A**) Pedigrees of families with mutations in the *IMPG2* gene. Patients with pathological findings are indicated by filled symbols. Family members with available clinical information and SD-OCT data [horizontal scans through the fovea shown in (**B**)] are marked with arrows, age in years is given below. A minor, dome-shaped foveal neuroretinal detachment filled with subretinal material above the RPE is observed for relatives in family 9-399 (OS > OD) and 8-553 (note the minor defects at the photoreceptor inner segment/outer segment junction (ellipsoid zone) in OD). Corresponding visual acuity (VA) results in logMAR are given below.

Carrier status of *IMGP2* mutations and ophthalmic findings were obtained for five individuals from four different families (Figure 5). The 29-year old asymptomatic son of index patient #3 (family 8-572, #3-1), had normal visual acuity and regular SD-OCT results consistent with his non-carrier status of the c.676G > T/p.(Glu226*) mutation of his mother. The sister of index patient #3 was reported to have a “similar” maculopathy but was not available for genetic or ophthalmic testing. The 11 year younger sister of #4 (family 9-399), age 44 (#4-1), carries the same heterozygous c.727G > C/p.(Ala243Pro) mutation in exon 7 of the *IMPG2* gene as her affected brother. Despite being asymptomatic, she had a slightly reduced visual acuity to 0.1 logMAR in her left eye due to a marked dome-shaped, foveal detachment of the neurosensory retina visualized by SD-OCT with some solid material above the seemingly intact RPE. A similar, but smaller lesion was observed in the SD-OCT scans of her right eye. Both asymptomatic sons of index patient #9 (family 9-274), age 40 (#9-1) and 42 (#9-2) respectively, inherited the c.3125A > G/p.(Tyr1042Cys) mutation in exon 15 of the *IMPG2* gene from their father. The older son showed a slight reduction of visual acuity to 0.1 logMAR in his right eye. SD-OCT scans revealed no abnormalities of the central retina in both brothers. Finally, patient #10 (family 8-553) has transmitted the c.3230G > T/p.(Cys1077Phe) mutation to her 31 year old son (#10-1) who complained about reading problems. Visual acuity was reduced to 0.2 logMAR in the right eye, corresponding to a minor, but noticeable dome-shaped foveal detachment with material above the RPE and defects at the photoreceptor inner segment/outer segment junction (ellipsoid zone) in SD-OCT. His left eye showed full visual acuity but SD-OCT revealed mild subfoveal accumulation of hyperreflective material without RPE or photoreceptor abnormalities. Taken together, SD-OCT findings in the offspring of patients with VMD that inherited the heterozygous *IMPG2* mutations from the affected relative indicate the very early subfoveal accumulation of debris that might progress to larger vitelliform lesions or atrophy later in life.

## 4. Discussion

In this study we have identified a series of novel mutations in the *IMPG1/IMPG2* genes and provide supportive evidence for their causativity in the development of vitelliform macular dystrophy. Patients with *IMPG1/IMPG2* mutations described here reveal strikingly similar phenotypic characteristics, including retinal lesions which can be divided into presumably consecutive stages: (i) singular vitelliform lesions in the central macula with detachment of the neurosensory retina in SD-OCT, with hyperreflective material located above the seemingly preserved Bruch’s membrane/RPE; (ii) resorption of the hyperreflective material leaving behind a dome-shaped, optically empty cavity; alternatively, the foveal cavity formed by retinal detachment may become successively filled with material; (iii) collapse of the cavity and central retinal atrophy with loss of RPE. This stage of disease lead to the most pronounced loss of visual acuity. 

Previous reports of clinical features in *IMPG*-associated VMD [7,8] are largely consistent with our findings including mild to moderate vision impairment, normal or borderline EOG and regular or slightly abnormal ERG findings as well as the appearance of the vitelliform macular lesions in color fundus images and FAF with subretinal material located strictly above the preserved RPE as depicted by SD-OCT. All our patients had bilateral singular, unifocal deposits without associated drusen-like lesions at the posterior pole. It has been proposed that satellite drusen-like lesions in the foveal zone are characteristic for *IMPG1*-linked VMD [8]. Since the ages of onset in the patients with satellite drusen ranged between 27 and 54 and the patients at examination were thus significantly older than our two patients with *IMPG1-*mutations (they are now in their teens), it remains to be seen whether they will also develop similar drusen-like abnormalities in adulthood. 

An earlier study reported on a c.713T > G mutation in exon 7 of the *IMPG1* gene in three families with autosomal dominant VMD [7]. Here, we have detected a similar mutation affecting c.713, a thymine to guanine exchange, in a simplex case of VMD. Both mutations lead to the substitution of the moderately conserved, hydrophobic amino acid leucine at position 238 to either a positively charged arginine [p.(Leu238Arg)] or to a cyclic proline [p.(Leu238Pro)]. Modelling of the SEAI domain of IMPG1 indicated that an arginine at position 238 destabilizes the protein [7] and mutations which result in an introduction of a proline are known to significantly affect protein stability [18]. Thus, the current finding of a second missense mutation affecting amino acid p.(Leu238) in a patient with VMD supports the causative role of defective IMPG1 molecules in disease via an as yet unknown dominant-negative effect or haploinsufficiency.

The second novel mutation in the *IMPG1* gene described in this study, a c.807 + 5G > A mutation in intron 7 causing aberrant splicing of the *IMPG1* mRNA, was found in a young Turkish boy in a homozygous state. Another splice mutation in intron 7, c.807 + 1G > T affecting the canonical GT donor splice sequence, has previously been described in a consanguineous Italian family [7]. Affected individuals of this family showed multifocal vitelliform deposits in addition to the prominent central lesion. Since the fundus images of these patients were taken at the age of 29 and 33, it will be interesting to observe whether our 13 year old patient will develop similar lesions when disease progresses with age. Interestingly, heterozygous carriers of the c.807 + 1G > T mutation show a slightly decreased EOG Arden ratio and tiny extramacular deposits in fundus examination suggesting that heterozygous carriers of the c.807 + 1G > T variant display a mild subclinical phenotype due to haploinsufficiency. Both heterozygous 35 and 40 year old parents of patient #2 are clinically asymptomatic with full visual acuity and regular findings in multimodal retinal imaging (data not shown). Our finding that a significant fraction of pre-mRNAs harboring the c.807 + 5G > A mutation are correctly spliced suggests that the level of functional IMPG1 is sufficient to prevent even subclinical abnormalities in heterozygous carriers. 

Homozygous or compound heterozygous nonsense, frameshift or splice site mutations in the *IMPG2* gene causing loss of IMPG2 function have repeatedly been described in patients with autosomal recessive retinitis pigmentosa (RP) (Figure 3, upper panel). These mutations are scattered throughout the gene but are preferentially found in the N-terminal part (23-276 aa), two central regions (530-560 aa and 758-805 aa) and the C-terminal part (964-1212 aa) of IMPG2. In addition, a homozygous p.(Phe124Leu) mutation was reported in a patient with mild maculopathy [5], a p.(Arg137Pro) mutation in a patient with Goldmann-Favre syndrome [19] and a heterozygous p.(Cys1077Phe) mutation in a patient with VMD [8]. These sequence variants as well as the stop and missense mutations found in our VMD patients are located in the N-terminal, the first central and C-terminal “hotspots”. The VMD-associated stop mutations p.(Glu226*), p.(Ser522*) and p.(Gln856*) in exon 7 and 13 of the *IMPG2* gene are likely targets for nonsense mediated decay (NMD) or lead to truncated proteins with impaired function and are therefore considered null mutations. So far, the evidence for a possible VMD causing effect of IMPG2 haploinsufficiency is based on the absence of the respective stop mutations in a large control population and the typical IMPG-linked phenotype of our three index patients. A recent report about patients with *IMPG2*-associated RP revealed that three quarters of the patients (n = 13) had profound macular abnormalities including bull’s eye maculopathy and macular atrophy [6]. These macular lesions are described as an early generalized loss of the outer retinal layers prior to RPE degeneration and are thus very different from the foveal subretinal deposits seen in our VMD patients. Nevertheless, it would be important to study the heterozygous parents of these and other *IMPG2*-associated RP patients for subtle ophthalmological abnormalities such as minor foveal deposits. 

The same c.3230G > T/p.(Cys1077Phe) *IMPG2* mutation detected in the 70-year old female index patient of family 8-553 has previously been reported in a 44-year old male with progressive bilateral vitelliform lesions and his asymptomatic 22-year old son [8]. SD-OCT scans of both of the son’s eyes revealed an abnormal thin hyperreflective line between the ellipsoid and outer segment-RPE interdigitation lines which was interpreted as a preclinical stage of *IMPG2*-VMD [8]. In support of an autosomal-dominant inheritance of the p.(Cys1077Phe) mutation, we observed distinct foveal lesions in the 31-year old son of patient #10 consisting with an accumulation of material above the RPE and mild visual impairment. Similarly, the sister of index patient #4 who is a heterozygous carrier of the familial c.727G > C/p.(Ala243Pro) mutation in *IMPG2* exon 7 and showed subclinical deposits above the RPE in the foveal zone and slightly decreased visual acuity. 

Both sons of family 9-274, who inherited the heterozygous c.3125A > G/p.(Tyr1042Cys) mutation in exon 15 of the *IMPG2* gene from their father, were 40 and 42 years of age when ophthalmologically examined. They were then asymptomatic and revealed regular retinal structures in SD-OCT. Their father was diagnosed with VMD not before the age of 62 and they may develop retinal disease later in life consistent with a rather late disease onset for symptomatic carriers of known C-terminal *IMPG2* missense mutations. 

Four of the five VMD-associated missense mutations in the *IMPG2* gene are located within a stretch of 80 amino acids in the two epidermal growth factor (EGF)-like motifs at the N-terminal vicinity of a highly hydrophobic putative transmembrane spanning domain. The p.(Gly1008Asp) amino acid exchange affects one of the four consensus sequences of glycosaminoglycan (GAG) attachment sites in IMPG2, namely Ser^1007^-Gly-(acidic) and may influence protein interaction that is important for the establishment and stability of the IPM matrix.

Six conserved cysteine residues in each of the two tandem EGF-like motifs of IMPG2 likely form the characteristic EGF-like disulfide bridges required for stability of EGF-like motifs [20]. IMPG2 mutations p.(Tyr1042Cys) and p.(Cys1077Phe) add or replace a cysteine residue in the EGF-like domain and may thus interfere with interchain disulfide bonding. Disruption of the disulfide bond pattern of EGF-like domains plays a major role in the pathogenesis of several inherited diseases [21,22]. Unravelling the molecular processes linking mutations in the *IMPG1* and *IMPG2* genes to retinal degeneration will be an important task for future experiments.

## 5. Conclusions

Taken together, this study supports the role of mutations in the genes encoding interphotoreceptor matrix proteins IMPG1 and IMPG2 in the development of vitelliform macular dystrophy and emphasizes the need to perform diagnostic testing of these genes especially in VMD patients without EOG abnormalities. Screening for larger structural alteration in the *IMPG1* and *IMPG2* genes by copy number variation (CNV) analysis might even increase the number of VMD cases associated with defects in these two genes. Finally, our report also highlights the importance of monitoring asymptomatic carriers for subclinical foveal abnormalities to further shed light on the inheritance pattern, penetrance and expressivity of *IMPG1/IMPG2* mutations.

## Figures and Tables

**Table 1:** Mutations in *IMPG1* and *IMPG2* in patients with vitelliform macular dystrophy.

Family ID	Patient ID	Gender	Exon	Nucleotide	MAF ^a^	Status	Effect	In Silico Analysis				Class	PMID
								*MutT* ^b^	*SIFT* ^c^	*PP2* ^d^	Splicing ^e^		
***IMPG1***													
13–501	#1	M	7	c.713T > C	-	het	p.(Leu238Pro)	dc (0.94)	del (0.01)	p (0.93)	-	lp	-
11–151	#2	M	7	c.807 + 5G > A	-	hom	splicing, p.?	n.a.	n.a.	n.a.	−20.6%	p	-
***IMPG2***													
8–572	#3	F	7	c.676G > T	-	het	p.(Glu226*)	n.a.	n.a.	n.a.	-	p	-
9–399	#4	M	7	c.727G > C	-	het	p.(Ala243Pro)	dc (0.9)	tol (0.07)	p (0.98)	-	lp	-
8–206	#5	M	13	c.1565C > G	-	het	p.(Ser522*)	n.a.	n.a.	n.a.	-	p	-
8–453	#6	M	13	c.2566C > T	-	het	p.(Gln856*)	n.a.	n.a.	n.a.	-	p	-
6–225	#7	F	15	c.3023G > A	8 × 10^−6^	het	p.(Gly1008Asp)	dc (1.0)	del (0)	p (0.99)	−8.7%	lp	-
6–75	#8	M	15	c.3047T > C	-	het	p.(Phe1016Ser)	dc (1.0)	del (0)	p (0.99)	-	lp	-
9–274	#9	M	15	c.3125A > G	-	het	p.(Tyr1042Cys)	dc (1.0)	del (0)	p (0.99)	-	lp	-
8–553	#10	F	15	c.3230G > T	-	het	p.(Cys1077Phe)	dc (1.0)	del (0)	p (0.98)	+3.6%	lp	25085631

PMID = pubmed ID; ^a^ Minor allele frequency (MAF) in total population, ExAC database in the public domain. ^b^ disease causing (dc), polymorphism (p). The probability value is given in brackets. A value close to 1 indicates a high ‘security’ of the prediction. ^c^ deleterious (del), tolerated (tol). Score ranges from 0 to 1. The amino acid substitution is predicted damaging if the score is ≤ 0.05, and tolerated if the score is > 0.05. ^d^ HumVar model, benign (b), possibly damaging (pd), probably damaging (p). ^e^ Splicing predictions at nearest natural junction, combines results from SpliceSiteFinder-like, MaxEntScan, NNSPLICE, GeneSplicer, Human Splice Finder.

**Table 2:** Summary of clinical information on patients with mutations in *IMGP1* and *IMPG2*.

Family ID ^a^	Patient ID	Age ^b^ [years]	Visual Acuity [logMAR]				Electrophysiology	
					EOG [Arden ratio]		Multifocal ERG	
			OD	OS	OD	OS	OD	OS
***IMPG1***								
13–501	#1	16 (12)	0.2	0.1	NA	NA	NA	NA
11-151	#2	6 (2)	0.1	0.1	1.6	5.0	normal	normal
		10	0.2	0.2	1.6	1.4	normal	normal
***IMPG2***								
8–572	#3	59 (57)	0.1	0.2	normal	normal	normal	minor reduction of amplitudes in ring 1
9–399	#4	54 (47)	0.4	0.2	2.3	2.3	NA	NA
8–206	#5	54 (46)	0.7	0.1	NA	NA	marked reduction of amplitudes in ring 1–2	marked reduction of amplitudes in ring 1–2
8–453	#6	64 (60)	0.6	0.2	1.9	1.8	NA	NA
6–225	#7	43 (33)	0.1	0.1	2.2	2.2	normal	normal
6–75	#8	57 (56)	0.1	0.2	NA	NA	minor reduction of amplitudes in ring 1	minor reduction of amplitudes in ring 1
9–274	#9	62 (62)	0.6	0.2	NA	NA	NA	NA
8–553	#10	70 (64)	0.4	0.3	2.5	4.1	marked reduction of amplitudes in ring 1–2	marked reduction of amplitudes in ring 1–2

logMAR = logarithm of the Minimum Angle of Resolution; EOG = electrooculogram; ERG = electroretinogram; OD = right eye; OS = left eye; NA = not available; ^a^ Patients have been sorted by ascending cDNA position of the respective mutation (see Table 1). ^b^ Age at last available examination (age at initial diagnosis).
